# Linear and Nonlinear Analyses of the Cardiac Autonomic Control in Children With Developmental Coordination Disorder: A Case-Control Study

**DOI:** 10.3389/fphys.2018.00267

**Published:** 2018-03-22

**Authors:** Jorge L. Cavalcante Neto, Antonio R. Zamunér, Bianca C. Moreno, Ester Silva, Eloisa Tudella

**Affiliations:** ^1^Núcleo de Estudos em Neuropediatria e Motricidade, Departamento de Fisioterapia, Universidade Federal de São Carlos, São Carlos, Brazil; ^2^Departamento de Kinesiología, Facultad de Ciencias de la Salud, Universidad Católica del Maule, Talca, Chile; ^3^Pró-Reitoria de Pesquisa e Pós-Graduação, Fisioterapia, Universidade do Sagrado Coração, Bauru, Brazil; ^4^Department of Physical Therapy, Universidade Federal de São Carlos, São Carlos, Brazil

**Keywords:** heart rate variability, autonomic nervous system, developmental coordination disorder, autonomic dysfunction, orthostatic stimulus, motor impairment

## Abstract

Children with Developmental Coordination Disorder (DCD) and children at risk for DCD (r-DCD) present motor impairments interfering in their school, leisure and daily activities. In addition, these children may have abnormalities in their cardiac autonomic control, which together with their motor impairments, restrict their health and functionality. Therefore, this study aimed to assess the cardiac autonomic control, by linear and nonlinear analysis, at supine and during an orthostatic stimulus in DCD, r-DCD and typically developed children. Thirteen DCD children (11 boys and 2 girls, aged 8.08 ± 0.79 years), 19 children at risk for DCD (13 boys and 6 girls, aged 8.10 ± 0.96 years) and 18 typically developed children, who constituted the control group (CG) (10 boys and 8 girls, aged 8.50 ± 0.96 years) underwent a heart rate variability (HRV) examination. R-R intervals were recorded in order to assess the cardiac autonomic control using a validated HR monitor. HRV was analyzed by linear and nonlinear methods and compared between r-DCD, DCD, and CG. The DCD group presented blunted cardiac autonomic adjustment to the orthostatic stimulus, which was not observed in r-DCD and CG. Regarding nonlinear analysis of HRV, the DCD group presented lower parasympathetic modulation in the supine position compared to the r-DCD and CG groups. In the within group analysis, only the DCD group did not increase HR from supine to standing posture. Symbolic analysis revealed a significant decrease in 2LV (*p* < 0.0001) and 2UV (*p* < 0.0001) indices from supine to orthostatic posture only in the CG. In conclusion, r-DCD and DCD children present cardiac autonomic dysfunction characterized by higher sympathetic, lower parasympathetic and lower complexity of cardiac autonomic control in the supine position, as well as a blunted autonomic adjustment to the orthostatic stimulus. Therefore, cardiovascular health improvement should be part of DCD children's management, even in cases of less severe motor impairment.

## Introduction

Developmental Coordination Disorder (DCD) is a disorder of motor coordination that significantly impairs the motor actions in children in different age groups (Harris et al., 2015). Current diagnostic guidelines for DCD involve a continuum of factors that address motor impairments in different children's life context (Blank et al., 2012; American Psychiatric Association, 2013). Due the complexity and difficulty in diagnosis, DCD prevalence around the world has varied from 1.7% (Lingam et al., 2009) to 24% (Valentini et al., 2017) among children of school age. Moreover, in addition to the terminology “children with DCD,” the term “children at risk for DCD” is also used in the literature, corresponding to the profile of children that present a motor impairment condition, but occupy the intermediate classification in standard motor tests (Smits-Engelsman et al., 2015; Wilson et al., 2017).

DCD related motor impairments contribute to restricting children's engagement with tasks involving accuracy and speed of movement (Licari et al., 2015; Wilson et al., 2017), which might cause fear of frustration and/or embarrassment (Cummins et al., 2005). Therefore, these children are more likely to choose solitary tasks and with more sedentary characteristics (Sylvestre et al., 2013). As a consequence, children with DCD have lower levels of habitual physical activity (Hendrix et al., 2014) tending to develop overweight and obesity (Cermak et al., 2015), thus increasing the risk for developing cardiovascular diseases (Rivilis et al., 2011).

Regarding cardiovascular risk assessment, the use of heart rate variability (HRV) analysis has been extensively applied to study the cardiac autonomic control in different populations and conditions, since it is a low-cost noninvasive tool providing important prognostic parameters for cardiovascular mortality, even in individuals without previous cardiovascular pathologies (Hillebrand et al., 2013; Wulsin et al., 2015).

Interestingly, some studies have reported autonomic dysfunction in subjects with motor disabilities (Hamamoto et al., 2003; Zamunér et al., 2011). However, studies assessing cardiac autonomic control in DCD are incipient. Coverdale et al. (2012) studied the cardiac autonomic control and baroreflex sensitivity in the resting supine condition in adolescents with DCD. The authors reported no differences between adolescents with probable DCD and healthy controls regarding linear HRV indices, but reported reduced baroreflex sensitivity, which was mainly attributed to an increased percentage of body fat. Chen et al. (2015) sought to study HRV in children at risk for DCD during cognitive tasks and concluded that they may show decreased HRV as a marker for altered ANS responses and potential deficits in the linkage between their perceptions and actions.

However, some points remain to be elucidated, such as the cardiac autonomic control adjustment to the action of standing up, which is a simple maneuver performed several times a day by children and requires fast and compensatory autonomic adjustments to maintain homeostasis during gravitational changes in the human cardiovascular system (Task Force of the European Society of Cardiology and the North American Society of Pacing and Electrophysiology, 1996). In addition, it is of interest to clarify whether the level of motor impairment (i.e., children with DCD vs. children at risk for DCD) is related to the severity of autonomic dysfunction. Moreover, heart rate (HR) modulation presents a nonlinear dynamic, which it is difficult to describe completely by linear methods (Signorini et al., 2001). Therefore, HRV nonlinear analyses have been applied and shown to provide complementary information about the underlying HR regulation mechanisms and to predict a pathological situation and/or a global depression of the organism (Goldberger, 1996; Guzzetti et al., 2005; Porta et al., 2009; Zamunér et al., 2015).

Elucidating whether children with DCD and at risk for DCD present abnormalities regarding cardiac autonomic control, may highlight the importance of a therapeutic approach aimed at improving cardiovascular health in this population. Therefore, this study sought to assess the cardiac autonomic control, by linear and nonlinear analysis, at supine and during an orthostatic stimulus in DCD, r-DCD and typically developed children. Our hypothesis is that children with DCD will present higher sympathetic, lower parasympathetic and lower complexity in the cardiac autonomic control in the supine position, as well as a blunted cardiac autonomic response to the orthostatic stimulus compared to typically developed children. Moreover, nonlinear analysis will provide complementary information to the linear analysis on cardiac autonomic control.

## Materials and methods

### Design and population

This was a cross-sectional, case-control study. All children were recruited from elementary schools in São Carlos, São Paulo, Brazil. Ninety-seven children were screened for eligibility according to the guidelines for DCD diagnostic based on the Diagnostic and Statistical Manual of Mental Disorders fifth edition (DSM-V) (American Psychiatric Association, 2013). According to the DSM-V, 25 children were classified as DCD, 32 children were classified as at risk for DCD (r-DCD) and 40 were classified as typically developed who were invited to take part in this study as controls. Out of this total, the parents of 47 children declined to participate in the study due the time mismatch. In the end, the final sample consisted of 13 DCD children (11 boys and 2 girls, aged 8.08 ± 0.79 years), 19 r-DCD children (13 boys and 6 girls, aged 8.10 ± 0.96 years) and 18 typically developed children that comprised the control group (CG, 10 boys, and 8 girls, aged 8.50 ± 0.96 years).

Children were included in the study if they satisfied the diagnostic criteria for DCD or r-DCD, based on DSM-V (American Psychiatric Association, 2013) and those with typical development were included in the CG. Exclusion criteria comprised a history of cardiovascular, respiratory, musculoskeletal, metabolic or neurological disorders, and continuous use of any medication.

All participants and their parents or guardians were informed as to the relevance of the research and about the experimental procedures. This study was carried out in accordance with the guidelines laid down in the Declaration of Helsinki. The protocol was approved by the Ethics Committee of the Federal University of Sao Carlos (number 47091115.0.0000.5504). All parents gave written informed consent with a verbal assent from the children.

### Instruments and procedures

The anthropometric profile of the children included body weight (kg), height (cm) and waist circumference (cm) assessments. Body weight was measured using an electronic scale (Type Welmy W110H; range 0.01–200 kg; precision 0.01 kg) linked to a stadiometer (Type Welmy W110H; range 60–200 cm; precision 1 mm), which was used to measure the height. Waist circumference (WC) was determined using a measuring tape (Wiso; range 0–200 cm; precision 1 mm). Body mass index (BMI) was calculated as body weight/height squared (kg/m2).

The children's motor performance concerning DCD identification was evaluated by the Movement Assessment Battery for Children–Second Edition (MABC-2). The MABC-2 is a standard instrument consisting of a set of eight motor tasks based on three motor domains: Manual Dexterity, Aiming & Catching and Balance to identify motor delay in children. According to the MABC-2 total scores, children were classified as: ≤56 points: children with significant movement delay (DCD children); from 57 to 67 points: children at risk of having movement delay (risk at DCD); and score above 67 points: children with no movement delay (typically developed children) (Henderson et al., 2007).

The children's general levels of physical activity were assessed using the Brazilian version of the Physical Activity Questionnaire for Children (PAQ-C) (Guedes and Guedes, 2015), which is a seven-day recall instrument. Responses were given on a five-point Likert scale. Each questionnaire item is scored between 1 (low) and 5 (high physical activity), and a mean score of all items constitutes the overall PAQ-C score. Higher values indicate better physical activity behavior (Kowalski et al., 1997). The PAQ-C was self-administered by the children's parents as secondary informants in a quiet room, since children under 10 years old often present difficulties remembering their previous daily activities (Silva and Malina, 2000). PAQ-C showed internal consistency values between 0.79 and 0.89 and test-retest reliability between 0.75 and 0.82. PAQ-C was previously validated using correlation analysis with the Godin and Shephard physical activity questionnaire (*r* = 0.41) and the Caltrac accelerometer (*r* = 0.39; Crocker et al., 1997).

### Experimental procedures

All experiments were carried out in the afternoon (13 p.m. to 18 p.m.) in order to minimize circadian changes. Room temperature was maintained at 22°C and relative air humidity at between 40 and 60%.

One week and the day prior to the cardiac autonomic control assessment, the children and their parents or guardians received relevant instructions to ensure a safe and satisfactory performance. Instructions were given to avoid the consumption of stimulating beverages or foods (e.g., coffee, soda, energy drinks, chocolate, black or green tea etc.,) and to suspend any major physical activity at least 24 h before the testing, to have a light meal before the testing and to have a good night's rest. All children were familiarized with the experimental protocol during a pilot test conducted 1 week prior to the study procedures.

### R-R intervals (RRi) recording

Children were subjected to the recordings of RRi in order to assess the cardiac autonomic control.

Upon arrival in the laboratory, the participants rested for about 20 min in supine posture for the HR and blood pressure to stabilize and to return to their baseline conditions. Then, RRi were recorded for 15 min in supine position and 10 min in orthostatic position (active standing) with spontaneous breathing. The participants' breaths per minute were recorded during the entire collection period by the evaluator, by visual inspection of thoracoabdominal movements. Participants who had a respiratory rate below 9 breaths per minute (0.15 Hz) would be excluded due to the fact that breathing influences the frequency bands of spectral analysis (Task Force of the European Society of Cardiology and the North American Society of Pacing and Electrophysiology, 1996). Participants were requested not to talk or move in order to avoid alterations and artifacts in the RRi.

RRi data were collected at a sampling rate of 1,000 Hz, using a validated HR monitor and a transmitter belt (Polar V800, Polar Electro Co. Ltda. Kempele, Finland) (Giles et al., 2016) placed in the thoracic region at the fifth intercostal space.

### HRV analyses

HRV was analyzed by linear and nonlinear methods using software developed by Dr. Alberto Porta from University of Milan (Montano et al., 1994; Porta et al., 2007). A region of 256 consecutive beats with the greatest stability in the RR time series was found for all children and in all conditions (i.e., supine and orthostatic postures), and was selected for HRV analyses.

#### Linear analysis

Spectral analysis was carried out by applying an autoregressive model in the previously selected RR section. The spectral components were obtained in low frequency (LF, 0.04–0.15 Hz) and high frequency bands (HF, 0.15–0.4 Hz) in absolute units (ms^2^). Normalized units were computed by dividing the absolute potency of LF or HF components by the total variance of RRi ($\sigma_{\text{RR}}^{2}$) minus the very low frequency component (0.003–0.04 Hz) and multiplying this ratio by 100.

#### Nonlinear analysis

The nonlinear methods used in the present study comprised symbolic analysis and Shannon entropy, both described in detail elsewhere (Porta et al., 2001).

Briefly, symbolic analysis comprises quantization of the RR time series selected for analysis in six uniformly distributed levels, where each beat receives a symbol (from 0 to 5). After that, four patterns are identified considering the sequences of three consecutive symbols: patterns without variation (0V), patterns with one variation (1V), patterns with two like variations (2LV), and patterns with two unlike variations (2UV). The percentage of each family's appearance is quantified. Previous studies (Guzzetti et al., 2001, 2005; Porta et al., 2001) have reported that the 0V% index represents sympathetic cardiac autonomic modulation, the 1V% represent both parasympathetic and sympathetic cardiac autonomic modulation, and the 2LV% 2UV% indices represent parasympathetic cardiac modulation.

Shannon entropy reflects the complexity of the RR time series by measuring the patterns' distribution complexity (sequences of three symbols). The presence of peaks (i.e., relevant patterns more frequently detected) or valleys (i.e., relevant missing or less frequent patterns) in the patterns' distribution determines the reduction of Shannon entropy. Conversely, maximal Shannon entropy is obtained when the patterns are identically distributed (Porta et al., 2001; Zamunér et al., 2015).

### Statistical analysis

Normality and homogeneity of variance assumptions were tested using the Shapiro-Wilk's and Levene's tests, respectively. Since several studies regarding DCD tend to group children with DCD and r-DCD, we performed a two-factor 2 × 2 mixed analysis of variance (ANOVA) with one between factor (CG vs. DCD and r-DCD grouped together) and one within factor (posture; supine vs. standing) to compare the differences between CG and DCD/r-DCD children in supine and orthostatic conditions. Following this, a two-factor 3 × 2 mixed ANOVA with one between factor (CG vs. DCD vs. r-DCD) and one within factor (posture; supine vs. standing) was performed to account for the severity of motor impairment. Where there was a significant interaction, analysis of the main effects was disregarded and the test for multiple comparisons with Bonferroni adjustment was performed. Assumptions for ANOVA were violated for $\sigma_{\text{RR}}^{2}$, LF and HF indices of HRV. Therefore, for these indices, between group comparisons and within group comparisons were performed using the Mann-Whitney and Wilcoxon tests, respectively, with Bonferroni adjustment *a priori*.

To control for a possible effect of confounding variables in the outcomes, a series of two-factor mixed analysis of covariance (ANCOVA) was computed considering gender, BMI, PAQ-C score and WC as covariates. Pearson's correlation coefficient (r) was used to assess the relationship between MABC-2 total scores and HRV indices. The significance level was set at 5%. Analyses were carried out using the SPSS (SPSS 22.0 version, Chicago, Illinois, USA).

## Results

### Demographic characteristics

Table 1 summarizes children's characterization by anthropometrical profile, motor performance assessed by MABC-2 total scores and levels of physical activity assessed by the PAQ-C scores in DCD, r-DCD, and CG. Significant difference between groups was only observed in MABC-2 total scores. As expected, CG presented higher MABC-2 total score compared to DCD (*p* < 0.01) and r-DCD (*p* < 0.01). Moreover, r-DCD presented higher MABC-2 total score compared to DCD (*p* < 0.01).

**Table 1:** Demographic characteristics of children with typical development (CG), children at risk for developmental coordination disorder (r-DCD) and children with DCD.

**Variables**	**CG (*n* = 18)**	**r-DCD (*n* = 19)**	**DCD (*n* = 13)**	**F value**	**p - value**
Gender (M/F)	10/8	13/6	11/2	-	0.23^*^
MABC-2 total score	75.61 (6.63)^#^^†^	62.70 (3.22)^†^	46.91 (9.82)	69.62	<0.001
Weight (kg)	33.58 (9.08)	33.30 (10.38)	38.81 (14.62)	1.05	0.35
Height (cm)	136.97 (7.75)	133.97 (9.60)	134.41 (7.85)	0.61	0.54
BMI (kg/m^2^)	17.74 (3.61)	18.22 (4.11)	21.01 (5.37)	2.27	0.11
WC (cm)	60.11 (10.17)	64.81 (11.73)	70.20 (12.10)	2.81	0.07
PAQ-C	2.91 (0.60)	2.46 (0.50)	2.46 (0.67)	2.69	0.07

Data are presented as mean (standard deviation). Between group comparisons were performed by one-way ANOVA and Tukey post hoc test;

* *chi-square test. MABC-2, Movement Assessment Battery for Children – Second Edition; BMI, Body Mass Index; WC, Waist circumference; PAQ-C, Physical Activity Questionnaire for Children*.

# p < 0.05 vs. r-DCD;

† *p < 0.05 vs. DCD*.

### HRV analysis

Results regarding interactions, main effects and multiple pairwise comparisons from the 2 × 2 ANOVA (i.e., considering DCD and r-DCD grouped together) are presented below and summarized in Table 2. Table 3 summarizes only the multiple pairwise comparisons provided by the mixed model 3 × 2 ANOVA, while interactions and main effects results are discussed below.

**Table 2:** Linear and nonlinear heart rate variability indices of children with typical development (CG), and children at risk for Developmental Coordination Disorder (r-DCD) and those with DCD grouped together.

	**CG (*****n*** = **18)**		**DCD/r-DCD (*****n*** = **32)**		***p*****-value**		
	**Supine**	**Standing**	**Supine**	**Standing**	**G**	**P**	**I**
HR (bpm)^a^	82 (11)	101 (14)^*^	89 (16)	99 (12)^†^	0.47	0.000	0.02
μRR (ms)^a^	750.1 (102.7)	601.3 (88.1)^*^	694.3 (107.13)	613.5 (73.4) ^†^	0.38	0.000	0.01
σ^2^RR (ms^2^)^b^^,^^c^	2496.0 (1715.5–9370.5)	2005.7 (1303.8–4071.6)^*^	3342.5 (2084.1–6168.8)	1586.2 (985.4–3087.1) ^†^	N/A	N/A	N/A
**SPECTRAL ANALYSIS**							
LF (ms^2^)^b^^,^^c^	673.0 (455.5–2056)	524.5 (309.5–931.5)^*^	1086 (476.7–2872.7)	598 (383.5–1353)^†^	N/A	N/A	N/A
HF (ms^2^)^b^^,^^c^	1572.5 (487.5–5265)	338.0 (164.5–509.7)^*^	1215.5 (707.7–3208)	397 (255.5–920.7)^†^	N/A	N/A	N/A
LF (nu)^a^	35.7 (15.6)	63.3 (15.7)^*^	42.7 (18.1)	58.3 (16.4) ^†^	0.82	0.000	0.01
HF (nu)^a^	64.3 (15.6)	36.7 (15.7)^*^	57.3 (18.1)	41.7 (16.4)	0.82	0.000	0.01
**NONLINEAR ANALYSES**							
SE^a^	3.92 (0.36)^#^	3.46 (0.30)^*^	3.34 (0.8)	3.31 (0.62)	0.02	0.03	0.048
**SYMBOLIC ANALYSIS**							
0V (%)^a^	11.5 (8.4)^#^	29.6 (11.2)^*^	25.5 (20.8)	31.1 (15.4)	0.04	0.000	0.02
1V (%)^a^	44.6 (7.6)	47.1 (4.0)	43.2 (10.8)	45.2 (7.2)	0.36	0.18	0.87
2LV (%)^a^	19.3 (6.7)^#^	12.0 (5.1)^*^	12.5 (7.9)	10.2 (6.5)	0.01	0.000	0.01
2UV (%)^a^	24.6 (13.0)	11.2 (5.9)^*^	18.7 (10.4)	13.5 (7.7)^†^	0.44	0.000	0.01

*Values are expressed as mean (SD) or median (1st; 3rd quartile). P, posture main effect; G, group main effect; I, interaction; HR, heart rate; μRR, mean of RR intervals; σ^2^RR, variance of RR intervals; LF, low frequency component of RR variability; HF, high frequency component of RR variability; nu, normalized units; SE, Shannon entropy; 0V, patterns with no variation; 1V, patterns with one variation; 2LV, patterns with two like variations; 2UV, patterns with two unlike variations; N/A, not applicable*.

* p < 0.05 CG supine vs. CG standing;

† p < 0.05 DCD supine vs. DCD standing;

# *p < 0.05 CG supine vs. DCD supine*.

a *Two-factor mixed ANOVA with Bonferroni adjustment a posteriori*.

b Mann-Whitney U test for between group comparisons;

c *Wilcoxon signed-rank test for within group comparisons*.

**Table 3:** Linear and nonlinear heart rate variability indices from children with typical development (CG), children at risk for Developmental Coordination Disorder (r-DCD) and those with DCD.

	**CG (*****n*** = **18)**		**r-DCD (*****n*** = **13)**		**DCD (*****n*** = **19)**	
	**Supine**	**Standing**	**Supine**	**Standing**	**Supine**	**Standing**
HR (bpm)^a^	82 (11)	101 (14) ^†^	86 (10)	100 (11) ^‡^	93 (22)	98 (12)
μRR (ms)^a^	750.1 (102.7)	601.3 (88.1)	706.7 (87.0)	607.6 (70.0)	676.2 (133.1)	622.1 (80.6)
σ^2^RR (ms^2^)^b^^,^^c^	2496.0 (1715.5–9370.5)	2005.7 (1303.8–4071.6) ^†^	3154.7 (2349.6–6222.8)	1584.1 (952.8–3052.9) ^‡^	3530.4 (1860–6503.8)	1588.3 (1031.9–4211.8) ^§^
**LINEAR ANALYSIS**						
LF (ms^2^)^b^^,^^c^	673.0 (455.5–2056)	524.5 (309.5–931.5) ^‡^	1260 (539–2985)	563 (381–1377) ^‡^	727 (448–2849.5)	633.0 (381–1569) ^§^
HF (ms^2^)^b^^,^^c^	1572.5 (487.5–5265)	338.0 (164.5–509.7) ^†^	1288 (768–3109)	312 (255–917) ^‡^	1150 (677–4118)	585.0 (274.5–1121.5) ^§^
LF (nu)^a^	35.7 (15.6)	63.3 (15.7) ^†^	42.1 (16.5)	57.9 (18.1)	43.6 (21.0)	58.8 (14.2) ^§^
HF (nu)^a^	64.3 (15.6)	36.7 (15.7) ^†^	57.9 (16.5)	42.1 (18.1)	56.3 (21.0)	41.2 (14.2) ^§^
**NONLINEAR ANALYSES**						
SE^a^	3.92 (0.36)	3.46 (0.30)	3.30 (0.83)	3.35 (0.55)	3.34 (0.80)	3.25 (0.73)
**SYMBOLIC ANALYSIS**						
0V (%)^a^	11.5 (8.4)	29.6 (11.2)	24.5 (19.5)	31.0 (14.5)	26.9 (23.3)	31.2 (17.3)
1V (%)^a^	44.6 (7.6)	47.1 (4.0)	44.9 (10.4)	46.1 (6.5)	40.8 (11.2)	43.9 (8.2)
2LV (%)^a^	19.3 (6.7)^*^^#^	12.0 (5.1) ^†^	12.7 (7.0)	9.8 (6.3)	12.3 (9.4)	10.7 (7.1)
2UV (%)^a^	24.6 (13.0)	11.2 (5.9) ^†^	17.8 (8.0)	13.0 (5.4)	20.0 (13.5)	14.2 (10.3)

*Values are expressed as mean (SD) or median (1st; 3rd quartile). HR, heart rate; μRR, mean of RR intervals; σ^2^RR, variance of RR intervals; LF, low frequency component of RR variability; HF, high frequency component of RR variability; nu, normalized units; SE, Shannon entropy; 0V, patterns with no variation; 1V, patterns with one variation; 2LV, patterns with two like variations; 2UV, patterns with two unlike variations*.

* p < 0.05 CG supine vs. r-DCD supine;

# p < 0.05 CG supine vs. DCD supine;

† p < 0.05 CG supine vs. CG standing;

‡ *p < 0.05 r-DCD supine vs. r-DCD standing*.

§ *p < 0.05 DCD supine vs. DCD standing*.

a *Two-factor mixed ANOVA with Bonferroni adjustment a posteriori*.

b *Mann-Whitney U test for between group comparisons*.

c *Wilcoxon signed-rank test for within group comparisons*.

#### Interaction between group (CG and DCD/r-DCD) and posture

A significant group × posture interaction was observed for HR [*F*_(1, 48)_ = 6.23; *p* = 0.02], μRR [*F*_(1, 48)_ = 8.03; *p* = 0.01], LFnu [*F*_(1, 48)_ = 7.05; *p* = 0.01], HFnu [*F*_(1, 48)_ = 7.05; *p* = 0.01], Shannon entropy [*F*_(1, 50)_ = 4.03; *p* = 0.049], 0V [*F*_(1, 50)_ = 5.17; *p* = 0.03], 2LV [*F*_(1, 50)_ = 5.26; *p* = 0.03], and 2UV [*F*_(1, 50)_ = 6.69; *p* = 0.01].

##### Between group multiple pairwise comparisons (CG vs. DCD/r-DCD)

Planned pairwise comparisons revealed that at rest in supine posture, DCD/r-DCD presented lower Shannon entropy (*p* = 0.01), lower 2LV (*p* = 0.004), and higher 0V (*p* = 0.01) compared to the CG. No significant differences were found between CG and DCD/r-DCD in supine posture for HR (*p* = 0.11), μRR (*p* = 0.09), and linear indices of HRV (LFnu, *p* = 0.18 and HFnu, *p* = 0.18).

Mann-Whitney test with a Bonferroni adjustment a priori revealed no significant differences between groups for σ^2^RR (*p* = 0.53), LF (*p* = 0.44), and HF (*p* = 0.76).

##### Within group multiple pairwise comparisons (supine vs. standing)

Regarding the comparisons between postures, both groups (CG and DCD/r-DCD) significantly (*p* < 0.05) decreased μRR and HFnu, and increased LFnu when moving from supine to standing. However, the CG also decreased Shannon entropy (*p* = 0.02) and 2LV (*p* < 0.0001) and increased 0V (*p* < 0.0001) indices, which was not observed in the DCD/r-DCD group (Shannon entropy, *p* = 0.75; 2LV, *p* = 0.10, and 0V, *p* = 0.10).

Wilcoxon test with a Bonferroni adjustment a priori showed that both groups decreased σ^2^RR, LF and HF during standing compared to the supine posture (*p* < 0.01).

#### Interaction between group (CG, r-DCD, and DCD) and posture

Table 3 summarizes the results accounting for the severity of motor impairment (i.e., DCD and r-DCD group stratified). There was a significant group × posture interaction for HR [*F*_(1, 47)_ = 4.30; *p* = 0.02], μRR [*F*_(1, 47)_ = 4.73; *p* = 0.01], LFnu [*F*_(1, 47)_ = 3.46; *p* = 0.04], HFnu [*F*_(1, 47)_ = 3.46; *p* = 0.04], 2LV [*F*_(1, 47)_ = 3.23; *p* = 0.048], and 2UV [*F*_(1, 47)_ = 3.23; *p* = 0.048].

Regarding Shannon entropy and 0V, no significant group × posture interaction [*F*_(1, 47)_ = 1.68; *p* = 0.20; *F*_(1, 47)_ = 2.42; *p* = 0.10; respectively] or main effect of group [*F*_(2, 47)_ = 3.07; *p* = 0.06; *F*_(2, 47)_ = 2.16; *p* = 0.13; respectively] was found. A significant main effect of posture was observed for the 0V index [*F*_(1, 47)_ = 11.91; *p* = 0.001]. Therefore, regardless of group, a significant increase in 0V pattern, reflecting cardiac sympathetic modulation, was observed when moving from supine to standing.

##### Between group multiple pairwise comparisons (CG vs. r-DCD vs. DCD)

Pairwise comparisons revealed no significant differences (*p* > 0.05) between groups in supine or standing postures for HR, μRR and linear indices of HRV (LFnu, HFnu). Regarding nonlinear analysis, CG presented higher 2LV compared to r-DCD (*p* = 0.03) and DCD (*p* = 0.046) groups in supine position, reflecting higher parasympathetic modulation in CG.

Mann-Whitney test with a Bonferroni correction a priori revealed no significant differences between groups for σ^2^RR, LF, and HF (*p* > 0.05).

##### Within group multiple pairwise comparisons (supine vs. standing)

Within group comparisons revealed significant increase in HR from supine to standing posture in CG (*p* < 0.001) and in the r-DCD group (*p* < 0.001) but not in the DCD group (*p* = 0.17). Symbolic analysis revealed a significant decrease in 2LV (*p* < 0.0001) and 2UV (*p* < 0.0001) from supine to standing in the CG, but not for the r-DCD (2LV, *p* = 0.11 and 2UV, *p* = 0.06) and DCD (2LV, *p* = 0.44 and 2UV, *p* = 0.06) groups.

All groups decreased σ^2^RR, LF, HF, HFnu, and increased LFnu during standing compared to the supine posture (*p* < 0.01).

#### Confounding factors

All results remained unchanged after performing an ANCOVA controlling for gender, BMI, PAQ-C score, and WC. Therefore, these results were not presented.

### Relationship between motor performance and cardiac autonomic control

**Figure 1:**
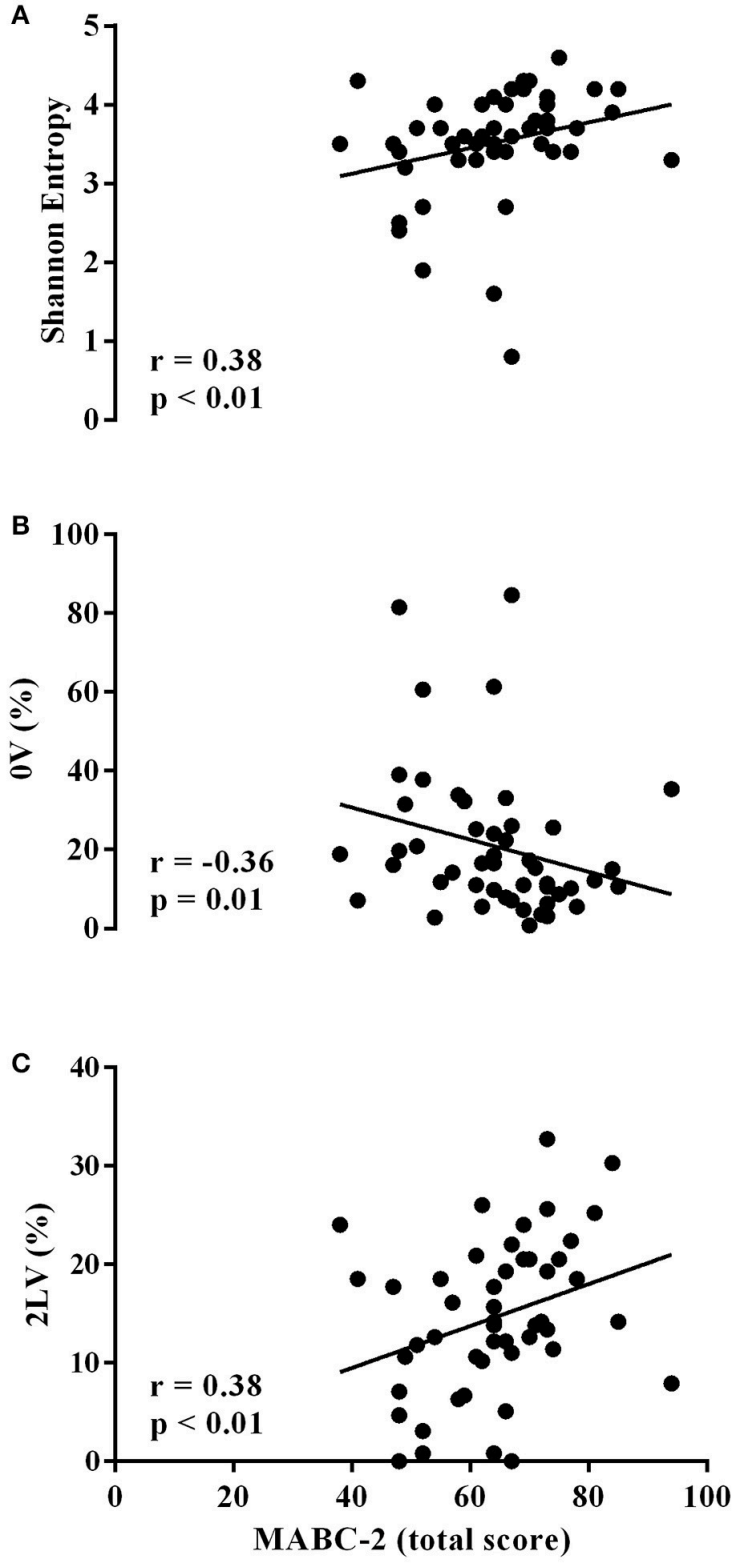
Scatter plots of Shannon entropy **(A)**, 0V pattern **(B)** and 2LV pattern of symbolic analysis **(C)** at rest on MABC-2 total score are shown. Each solid circle is relevant to a pair (MABC-2, nonlinear heart rate variability indices) computed over a single individual.

Correlation analysis revealed a significant association between MABC-2 total score and Shannon entropy (*r* = 0.38; *p* < 0.01; Figure 1A), 0V (*r* = −0.36; *p* = 0.01; Figure 1B) and 2LV (*r* = 0.38; *p* < 0.01; Figure 1C) assessed at supine posture.

## Discussion

The main findings of the present study were that: (1) DCD and r-DCD children grouped together presented higher sympathetic, lower parasympathetic, lower complexity of cardiac autonomic control in supine position and blunted autonomic adjustment to the orthostatic stimulus compared to typically developed children; (2) when stratified, r-DCD and especially the DCD group, presented blunted cardiac autonomic adjustment to the orthostatic stimulus, which was not observed in the CG group, reflecting that the severity of motor impairment might be related to the severity of autonomic dysfunction; (3) significant relationships between MABC-2 total score and nonlinear indices of HRV were observed, suggesting that the lower the motor performance the lower the complexity of the cardiac autonomic control (Shannon entropy) and the cardiac parasympathetic modulation (2LV) and the higher the cardiac sympathetic modulation (0V); and (4) nonlinear analysis of HRV provided non-redundant and complementary information about cardiac autonomic control, depicting cardiac autonomic abnormalities not identified by traditional linear methods, even in children with less severe motor impairment (i.e., r-DCD).

To the best of our knowledge, this is the first study addressing cardiac autonomic control by linear and nonlinear analysis of HRV in children with DCD and r-DCD. However, previous studies have addressed cardiovascular variables in this population (Chirico et al., 2012; Coverdale et al., 2012; Chen et al., 2015). Coverdale et al. (2012) studied the cardiac autonomic control and baroreflex sensitivity in the resting supine condition in adolescents with suspect and probable DCD. Despite being a different population, our results partially corroborate their findings. The authors (Coverdale et al., 2012) reported no significant differences in cardiac autonomic control, quantified by linear HRV indices, between controls and suspect and/or probable DCD groups in supine position. On the other hand, baroreflex sensitivity was lower in the probable DCD group compared to the CG and suspect DCD, which was mainly attributed to the higher body fat percentage in this group. Chirico et al. (2011) aimed to compare the heart left ventricular structure and function between children with DCD and healthy controls. The authors reported significantly elevated end-diastolic volume, diastolic chamber size, stroke volume, and cardiac output in children with probable DCD, suggesting obesity related changes in the left ventricle. Moreover, Chirico et al. (2012) found that elevated fat mass in adolescents with probable DCD contributes to higher cardiac output and left ventricle mass over time compared to typically developed controls. Nevertheless, in the present study the CG was matched to the DCD group for BMI. Therefore, anthropometric characteristics, such as BMI, weight and WC, were not different between groups. Thus, obesity may not be the only factor explaining the current results.

Another interesting study in this field was carried out by Chen et al. (2015). The authors aimed to study cardiac autonomic control in children with or at risk for DCD during cognitive tasks at different levels of difficulty. The authors found higher cardiac sympathetic modulation in children with DCD in comparison to typically developed children. In addition, the authors reported a blunted cardiac autonomic adjustment to some cognitive tasks in the DCD group compared to controls. These results are in agreement with our findings, since a blunted cardiac autonomic response, characterized by limited decrease in parasympathetic, as observed by the indices 2LV and 2UV, and limited increase in sympathetic cardiac modulation, as observed by the 0V index, were also observed in DCD and r-DCD groups during gravitational stimulus. The authors suggested that the higher cardiac sympathetic modulation in DCD children might be due to lower levels of aerobic fitness. In the present study, although not statistically significant, r-DCD and DCD groups presented lower levels of physical activity, as assessed by the PAQ-C. However, the results remained unchanged after considering the level of physical activity as covariate. Therefore, some hypothesis other than obesity and aerobic fitness underlying the present results should also be considered.

One possible explanation might be related to the patterns of connectivity and neural recruitment observed in children with DCD. Wilson et al. (2017) revised systematic neuroimaging data from studies performed with DCD and stated that the neural activity of these children is similar to that observed in children with mild cerebral palsy and born preterm. Indeed, several studies have reported cardiac autonomic abnormalities in those born preterm (Clairambault et al., 1992; van Ravenswaaij-Arts et al., 1995) and in children with cerebral palsy (Park et al., 2002; Zamunér et al., 2011; Amichai and Katz-Leurer, 2014), characterized by greater sympathetic, lower parasympathetic and lower complexity in cardiac autonomic modulation, compared to typically developed children. In addition, impaired autonomic adjustment to the postural changes was also observed in children with cerebral palsy (Park et al., 2002; Zamunér et al., 2011), suggesting that sympathetic activation was not enough to overcome the orthostatic stress imposed on these children. These findings corroborate our results.

Moreover, Zamunér et al. (2011) reported that the more severe the child's motor impairment, the lower the HRV, which was also found in the present study, since cardiac autonomic dysfunction was more pronounced in the DCD group than in the r-DCD group. In addition, significant relationships between motor performance and nonlinear HRV indices were also observed. Regarding cerebral palsy, the authors justified their results suggesting a possible effect from the loss of hemispherical influences on autonomic control resulting from existing cerebral lesions. Indeed, some authors suggest that in children with DCD the neural substrate mimics that of cerebral palsy (Peters et al., 2013) with lower brain activity in some cortical areas (Querne et al., 2008; Kashiwagi et al., 2009) responsible for body adjustment functions, which also could account for abnormalities in cardiac autonomic control and its relationship with the severity of motor impairment in these children. Therefore, future studies should address whether there is a relationship between neuroimaging data, neural activity and autonomic dysfunction in DCD children.

Another noteworthy result was that nonlinear analysis of HRV provided relevant complementary information about cardiac autonomic regulation, identifying cardiac autonomic abnormalities not detected by traditional linear methods, even in children with less severe motor impairment (i.e., r-DCD). Nonlinear methods have been shown to better describe nonlinear dynamics in RRi time series than linear methods (Voss et al., 1996; Zamunér et al., 2013, 2015), thus providing additional insights regarding cardiac autonomic regulation. Shannon entropy's analysis revealed that r-DCD/DCD children presented reduced complexity in cardiac autonomic regulation. Several studies have reported that a decrease in complexity indices might represent a depressed organ function, a loss of interaction between subsystems, an overwhelming action of a subsystem over others and an impairment of regulatory mechanisms, therefore constituting a clear hallmark of a pathological condition (Porta et al., 2009; Zamunér et al., 2013, 2015). Furthermore, symbolic analysis enabled the quantification of nonreciprocal changes in sympathetic (0V pattern) and parasympathetic (2LV and 2UV patterns) cardiac autonomic control, differently from spectral analysis, which provides more information regarding the parasympathetic branch (Porta et al., 2009).

Despite these interesting results, some study limitations need to be acknowledged. It is worth mentioning that we observed significant group × posture interaction for HR, revealing a blunted increase of HR to the orthostatic stimulus in the DCD group. It is well known that linear and some nonlinear indices of HRV are significantly correlated with average HR (Sacha and Pluta, 2008; Sacha et al., 2013; Bolea et al., 2016), including in pediatric population (Gasior et al., 2015). Therefore, several mathematical procedures have been proposed in order to attenuate the HRV dependence on HR (Sacha et al., 2013; Monfredi et al., 2014; Bolea et al., 2016). Nevertheless, to the best of our knowledge, no procedures have been proposed to normalize the nonlinear indices used in the present study (i.e. Shannon entropy and symbolic analysis indices). Thus, HRV normalization was not performed. However, a possible influence of HR should not be a bias on the present study due to no significant differences between groups regarding HR. Moreover, HRV indices, specially the nonlinear ones, provided additional information to the HR itself. Even though, it is important to highlight that future studies should quantify these nonlinear indices dependence on HR and propose methods to address this issue.

Another limitation of the present study was that from the 97 children screened for eligibility, 48% of the parents declined to participate in the study, which may have led to a possible sample selection bias. Even though, this is an important outcome drawing attention to the need to improve parents' awareness and education about DCD. Moreover, the difference between groups regarding gender should be mentioned. Although it was not significant and results remained unchanged after considering it as covariate, future studies should address this issue. Furthermore, 24-h HRV should also be considered in future studies in order to elucidate possible cardiac autonomic control abnormalities in this population during daily life and sleep.

In conclusion, r-DCD and especially DCD children presented higher sympathetic, lower parasympathetic, lower complexity of the cardiac autonomic control in supine position and blunted autonomic adjustment to the orthostatic stimulus compared to the typically developed children. In addition, the lower the motor performance, the lower the complexity of the cardiac autonomic control and the cardiac parasympathetic modulation; and the higher the cardiac sympathetic control. Thus, since the assessment of cardiac autonomic control is easily performed by noninvasive method and provides an important parameter for cardiovascular risk prognosis, it should be part of routine assessments in this population.

## Author contributions

JC, AZ, ES, and ET designed the study. JC and AZ performed the experiments and drafted the manuscript. JC, AZ, and BM analyzed the data. JC, AZ, BM, ES, and ET interpreted the data. JC, AZ, BM, ES, and ET revised the manuscript. JC, AZ, BM, ES, and ET approved the final version of the manuscript to be published.

### Conflict of interest statement

The authors declare that the research was conducted in the absence of any commercial or financial relationships that could be construed as a potential conflict of interest.
